# Contributions to Knowledge of the *Dictyocaulus* Infection of the Red Deer

**DOI:** 10.3390/vetsci12060595

**Published:** 2025-06-17

**Authors:** M. González-Velo, A. Espinosa-Sánchez, A. Ripa, M. A. Hurtado-Preciado, M. A. Habela Martínez-Estéllez, J. L. Fernández-García, C. Bazo-Pérez

**Affiliations:** 1 Parasitology and Animal Health Department, Veterinary School, University of Extremadura, 10003 Cáceres, Spain; mgonzalechj@alumnos.unex.es (M.G.-V.); mahabela@unex.es (M.A.H.M.-E.); cristinabazoperez@gmail.com (C.B.-P.); 2 Genetics and Animal Breeding Department, Veterinary School, University of Extremadura, 10003 Cáceres, Spain; aespinosa84@unex.es (A.E.-S.); arlb.vet@gmail.com (A.R.); hurtado728@yahoo.es (M.A.H.-P.)

**Keywords:** COI-barcoding, *Dictyocalulus*, morphological and molecular identification, red deer

## Abstract

Dictyocaulosis is a parasitic disease that affects ungulate species, including red deer (*Cervus elaphus*). The genus *Dictyocaulus* comprises eighteen species, but only four have been reported to infect red deer. The disease is characterized by respiratory tract infection, particularly in the lungs, bronchi, and bronchioles, leading to inflammatory and hemorrhagic microscopic lesions, as well as emphysema and edema. The biological cycle involves a female ovipositing larvated eggs in the bronchi and trachea, which are expelled to the exterior through coughing or feces, releasing L1 into the environment. In this study, 106 adult red deer were collected from seven locations in Extremadura (Spain). Eight positive lungs were initially assessed by morphological identification, revealing a mean intensity of 13.3 adult worms per infected lung, with a global decrease to an average of 1.8 adult worms per sampled lung. The presence of adult worms in the upper and middle respiratory tract was confirmed through anatomopathological analysis. Molecular identification was performed by sequencing the COI gene. The results indicated the presence of three genetic groups, supported by significant subdivision using the *ɸ_ST_* measure. *D. cervi* and *D. viviparus* exhibited their respective matrilineal ancestry, while *D. eckerti* and *D. cervi* demonstrated matrilineal sharing. Consequently, the possibility of introgression between these two species was suggested. Although *D. viviparus* had previously been identified in the same Spanish region based on morphological characteristics, *D. cervi* and *D. eckerti* were reported for the first time in the explored geographic area.

## Simple Summary

The genus *Dictyocaulus* comprises eighteen species of worms, but only four of these infect red deer. Infection results in damage to the respiratory tract, ranging from emphysema or edema to microscopic inflammatory and hemorrhagic lesions. Larvated eggs are expelled externally through coughing or feces, leading to the release of L1 into the environment. Worms from positive lungs collected in Extremadura (Spain) were examined using morphological identification, along with analyses of anatomopathological lesions and molecular barcode identification. The presence of three genetic groups was confirmed through significant subdivision using the *ɸ_ST_* measure, but *D. cervi* and *D. viviparus* exhibited their respective matrilineal ancestry, while *D. eckerti* and *D. cervi* showed matrilineal sharing. Consequently, the need to evaluate introgression between these two species was highlighted. *D. viviparus* was discarded despite having been previously reported in the same Spanish location using morphological methods, and *D. cervi* and *D. eckerti* were identified for the first time in the geographical area explored.

## 1. Introduction

Throughout the history of humankind, attention has been paid to the study of zoonoses and how wildlife has been involved in health alerts, which includes transmissible diseases to livestock, as is the case in tuberculosis, protozoa, or helminths such as *Dictyocaulus* [1,2]. Helminth parasitic diseases are among those of high importance for wild ruminants’ dynamics, but four genera, *Ascaris*, *Dictyocaulus*, *Strongyloides*, and *Trichuris* demand special attention for health and husbandry in ruminants [3]. *Dictyocaulus* species (Nematoda: Trichostrongyloidea) are distributed worldwide, infecting both even-toed ungulates—*D. viviparus*, *D. filaria*, *D. eckerti*, *D. murmanensis*, *D. africanus*, *D. capreolus*, *D. cervi*, and *D. cameli*—and odd-toed ungulates—*D. arnfieldi* and *D. pandionis*—within Artiodactyla mammals.

The genus *Dictyocaulus* (Nematoda: Dictyocaulidae) was described by Railliet and Henry (1907) for the first time based on morphological traits [4]. Advances in optical microscopy and scanning electron microscopy from the end of the 19th century into the 20th century furthered the development of histological techniques and allowed more accurate morphological identification of these nematodes [5], improving the species taxonomic classification of distinct species, as in the case of *Dictyocaulus capreolus* [6]. Despite this, most species of the genus *Dictyocaulus* are still identified by the parasitized host, even in the 21st century [3]. In Spain, *Dictyocaulus viviparus* and *Dictyocaulus filaria* have been reported in domestic ruminants [7,8]. However, some reports acknowledged major difficulties for the identification of species occurring in wild ruminants, due to the high level of morphological similarity exhibited among several species [9]. In this respect, prior to the advent of molecular identification, all infections of lungworms from red deer were identified as *D. viviparus* which parasitized cattle. However, the molecular characterization of *Dictyocaulus* spp. has revealed the existence of several distinct clades or species [2,3,8,10,11,12] and has also been of value in estimating phylogenetic relationships among trichostrongyloid and metastrongyloid nematodes [7,13]. Although the genus *Dictyocaulus* belongs in the monotypic family Dictyocaulidae with eighteen nominal species, only five species have been confirmed to be valid based on molecular genetic data: *D. viviparus*, *D. filaria*, *D. eckerti*, *D. capreolus*, and *D. cervi* ([3] and references therein).

These parasites are found in the small and large airways of the host, potentially causing parasitic bronchitis (dictyocaulosis), sometimes a fatal disease, especially in cattle, sheep, and farmed red deer [14]. Under this scenario, a breakthrough for the development of control strategies to prevent infection by *Dictyocaulus* was a topic of concern because different species of cervids carrying these parasites behave as true vectors to livestock [1,15,16]. In this regard, relevant research has provided control strategies based on vaccine preparations with an effective immunological response against these parasites [17,18]. However, it was noted that the nematodes became more resistant to the former vaccines, which is the reason why repeated improvement was achieved until the “Bovilis Huskvac” vaccine became available, offering a 95–98% level of protection [19]. Unfortunately, this vaccine against pulmonary worms is not widely used despite its usefulness. Practical concerns, such as a short shelf life and the availability of anthelmintics with persistent efficacy against *Dictyocaulus*, have apparently made vaccination a less attractive control option [19]. Recently, it has been described that resistance to antiparasitic drugs, such as fenbendazole and albendazole [20] and macrocyclic lactones [21] in cattle, has reactivated interest in these parasites. All lungworms were reported as *Dictyocaulus viviparus* in feral deer species [22,23], and a few studies on native Iberian red deer in Extremadura (Spain) suggested a low level of infection [24]. However, recent research advocates for investigating the full host range, epidemiology, potential impacts, and cross-transmission events with livestock of *Dictyocaulus* spp. with special attention paid to cervids worldwide [14,25], because the levels of infection with lungworms in free-ranging deer remain largely unknown [8,14,25,26]. In addition, global opinion suggests that objective methods of species-specific identification should be recommended [3], as cross-transmission has not yet been described [27]. For all these reasons, this report aims to contribute to the knowledge of *Dictyocaulus* species by examining aspects such as morphology, prevalence, and associated lesions, with a primary focus on conducting a genetic analysis using DNA molecular techniques, to achieve precise species-specific identification of the parasite responsible for dictyocaulosis in free-ranging red deer in Extremadura, southwestern Spain.

## 2. Materials and Methods

**Figure 1:**
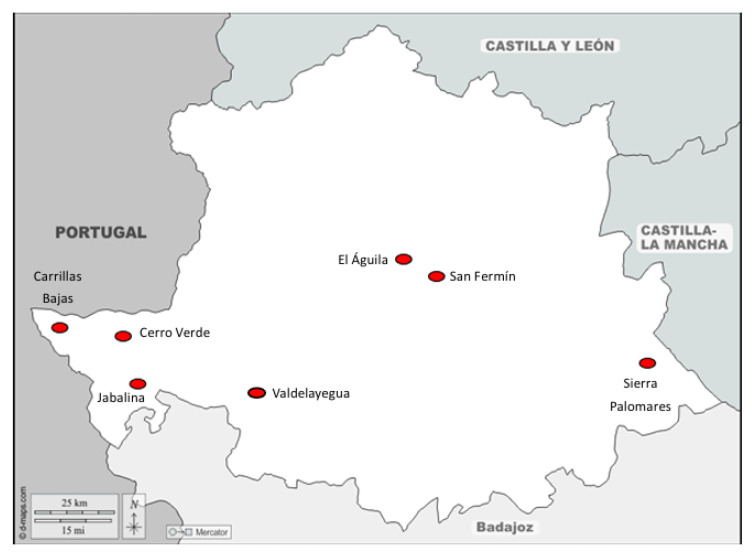
Map of areas of collection in north Extremadura.

The lungs of 36 deer hunted in seven enclosures from the province of Cáceres were studied (Figure 1). Table 1 shows the enclosures/estates and the dates when hunting activities were carried out for sampling. Carcass inspection was performed by a dedicated official veterinarian (Junta de Extremadura, Badajoz, Spain) following the regulation (EC) No. 853/2004 of the European Parliament and the executive regulation (EU) 2015/1375. Accordingly, 5 to 6 lungs, along with their respective tracheas, were collected at each site. Each lung was individually stored in an airtight bag and transported at 4 °C to ensure proper preservation.

### 2.1. Necropsies and Microscopic Examination

The lungs, previously squeezed, were opened from the trachea to the bronchi in a specialized laboratory of the Hospital Clínico Veterinario (University of Extremadura, Badajoz, Spain) using appropriate preventive measures. The presence of worms was verified by eye. After, macroscopic worms were introduced in a propylene bottle containing 96% Ethanol and stored at 4 °C until processing. Finally, morphological traits were assayed following keys under a NIKON H550S (Tokyo, Japan) light microscope [5,28], focusing on the shape of the anterior end (mouth and esophagus) and on the spicules of males. In any case, the lungs were also subjected to complementary examinations by slide scraping of the trachea and bronchi for eggs and/or larvae, which were subsequently examined by light microscopy. Of the seven enclosures sampled, only five had available and positive lungs. On the one hand, only larvae were observed after histological analysis in San Fermin. On the other hand, all lungs were mistakenly used for pathological anatomy in Cuadrillas bajas. Consequently, both positive enclosures were rejected for further analysis. Ultimately, three sites with positive lungs—Sierra Palomares, Jabalina, and Cerro Verde—were selected for molecular studies, maintaining a balanced sample size of five worms each. All data were recorded in an Excel sheet. Statistical agreement between macroscopic and microscopic findings was assessed using Cohen’s Kappa statistic in SPSS 15.0 (under UNEX License).

### 2.2. Anatomopathological Study

The macroscopic and microscopic lesions caused by worms were examined. In particular, affected areas of lungs that tested positive were embedded in formalin. Subsequently, tissue slices were processed using a conventional hematoxylin and eosin histopathological technique.

### 2.3. Molecular Procedures

#### 2.3.1. DNA Extraction

A total of 15 worms were processed, with five collected from each positive-lung enclosure. Samples from Sierra Palomares (SP), Jabalina (JB), and Cerro Verde (CV) were selected for molecular analysis. To ensure sterility and prevent cross-contamination, half a centimeter of the medial portion of adults—excluding genitalia—or larvae of the parasites was preserved for DNA extraction in a dedicated room. The DNA template was purified using a modified salting-out protocol [29], incorporating Zymo-Spin II C columns, and subsequently eluted in 400 μL of molecular biology-grade water.

#### 2.3.2. End-Point PCR and Primers

End-point PCR was performed using primers that target a specific portion of the COI mitochondrial gene as follows: COX I_F (5′-TTTTTTTTGGGCATCCTGAGGTTTAT-3′) and COX I_R (5′-TAAAGAAAGAAAGAACATAATGAAAAAATG-3′) [30]. The PCR master-mix (MM) was prepared following published protocols [31]. Briefly, 10 μL of each 10× NH4 buffer, 2 mM dNTPs, 10 μM COX I_R and F primers, and 3 μL MgCl2 (50 mM) were incorporated up to 90 μL with molecular biology-grade water. Individual assays were conducted using 18 μL MM and 2 μL of the DNA template. The thermocycler conditions were as follows: Pre-Denaturation at 95 °C for 5 min; 35 cycles at 94 °C for 60 s, 50 °C for 60 s, 72 °C for 60 s, and final extension at 72 °C for 7 min. PCR products were visualized by 1.6% (*w*/*v*) agarose electrophoresis SYBR safe dye. A 100 bp molecular weight ladder was used to monitor amplicon size under a 312 nm ultraviolet transilluminator.

#### 2.3.3. Sequencing, Alignment, and Comparison Through Phylogenetic Networks

The PCR products (5 μL) were purified with Ex-Spure (NimaGen, Nijmegen, The Netherlands) following the manufacturer’s recommendations and then diluted to 10 μL with ultrapure water. The purified PCR templates (5 µL) were sequenced using the Big Dye^®^ ver. 3.1 cycle sequencing kit (Thermo Fisher Scientific Waltham, MA, USA), with residuals removed via Performa^®^ DTR cartridges (Edge Bio, San Jose, CA, USA). Sequencing profiles were obtained through capillary electrophoresis using the Applied Biosystems™ 3130 DNA Analyzer (Waltham, MA, USA) and analyzed with ABI Sequencing Analysis ver. 5.2 software (Applied Biosystems, Waltham, MA, USA).

To identify species within the genus *Dictyocaulus*, sequences from the GenBank database (NCBI, USA) were filtered. The search was restricted to 250 sequences that matched or exceeded the coverage of the COI segment analyzed in this study. A similarity cutoff of ≥90% was applied to select sequences belonging to *Dictyocaulus* [32]. The last species downloaded were *Arthrosoma* spp. after filtering under this criterion. Thirty sequences among those downloaded belonged to the genus *Dictyocaulus*, with 100% coverage (320 bp) with respect to the 13 sequences from this study. Consequently, a total of 115 sequences were selected, with 102 from GenBank (NCBI, USA). These sequences were collapsed into haplotypes using DnaSP ver 6.1 [33]. Haplotypes were then used to create a standard Median-Joining (MJ) network [34] of genetic relationships in the program PopArt v. 1.7. (Population Analysis with Reticulate Trees) [35]. Once the haplotypes of the genus *Dictyocaulus* were identified, the genetic relationships were exclusively redrawn using these haplotypes and positions were visualized on a physical map using GPS data. Divergence among a phylogenetic cluster was assessed using the fixation index statistic (*ɸ_ST_*) using the grouping of sequenced data as follows: (1) *D. cervi*, (2) *D. Eckerti*/*D.cervi*, and (3) *D. vivparus* in PopArt v. 1.7. The sequences were deposited in the GeneBank database (NCBI, USA) under the accession numbers PV700498–PV700500.

## 3. Results

### 3.1. Prevalence of Infection by Dictyocaulus in Deer at Sampling Location

Table 2 records the number of positive animals and the number of adult worms in each of the 36 lungs processed.

The frequency of infected lungs was 13.9% after the macroscopic analysis but there was an average adult count of 12.8 (SD 7.1) (rank 4–22). However, higher frequency was found after microscopic analysis with 22.2% of lungs being positive. Although at first glance Cohen’s Kappa test suggested substantial agreement (κ = 0.72) between the macroscopic and microscopic analyses with a significant *p*-value (*p* < 0.001), there was not perfect agreement, indicating some discrepancies. Furthermore, the mean value for adult worms suggested large variability in the count of adult parasites per infected red deer. Despite the globally calculated average abundance of worms across all lungs being relatively low—yielding an average of 1.8 adult worms per lung (SD = 5.1)—it is relevant to highlight that 71.4% of the enclosure exhibited varying signs of parasitism. Furthermore, the results highlight the relevance of the microscopic analysis over the macroscopic inspection because larvae are much more common in lungs than macroscopically observable adult worms.

### 3.2. Morphological Identification and Anatomopathological Findings in Dictyocaulus Infection

**Figure 2:**
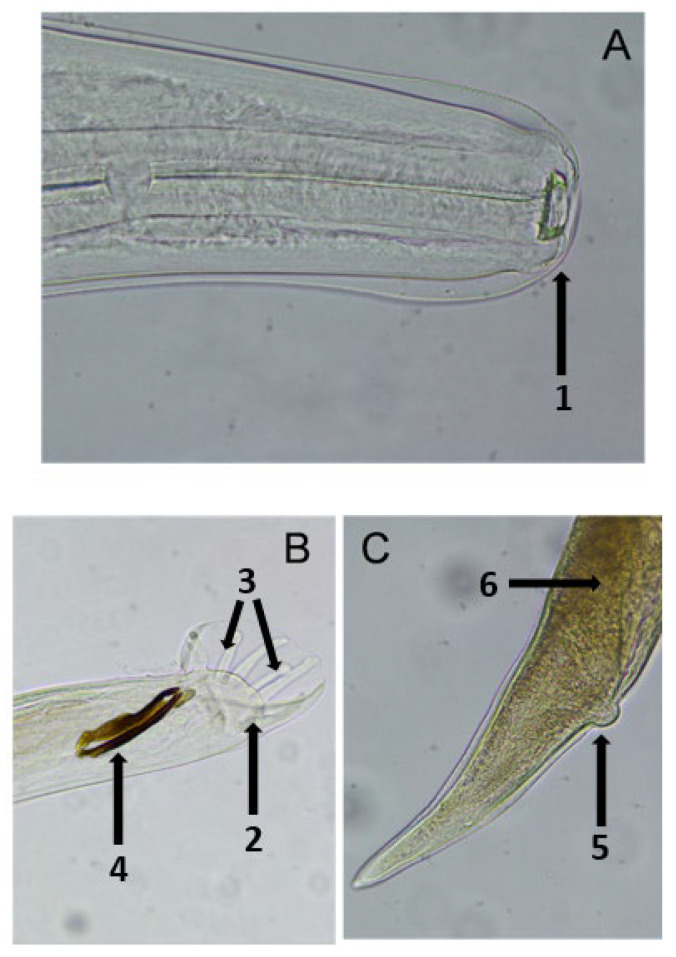
(**A**) Anterior end of *Dictyocaulus* spp.: (1) mouth with a well-pronounced cuticular ring. (**B**) Posterior end of male: (2) bursa copulatrix; (3) supporting ray; (4) spiculae. (**C**) Posterior end of female: (5) anus region; (6) uterus with larvated eggs.

Following the collection, preservation, and preparation of worms for microscopic observation, adult specimens were meticulously examined for accurate identification. It was determined that the buccal capsule was small, oval-shaped, and encircled by simple lips (Figure 2A), as expected. Additionally, morphological differences between males and females were analyzed. The male exhibited an expanded cuticle with a series of supporting rays forming the copulatory bursa, along with spicules that facilitated copulation. In contrast, the female’s posterior end was conical, featuring a small, rounded protrusion known as the anal region (Figure 2B,C). Notably, the uterus of the female was filled with eggs, specifically larvae in the L1 stage (Figure 2C).

**Figure 3:**
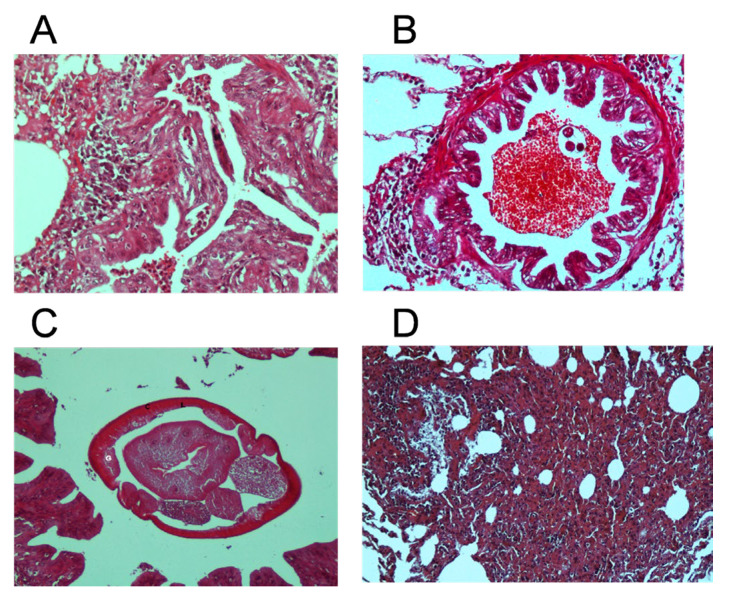
(**A**) Bronchiolitis with bronchiolar epithelial destruction. (**B**) Transversal histological section of an adult *Dictyocaulus* spp. within the bronchial lumen, surrounded by areas of hemorrhage. (**C**). Histological cross-section of an adult *Dictyocaulus* spp. within the bronchial lumen, highlighting key structural components: the fibrous capsule (**C**), acellular laminated membrane (L), and germinative membrane (G). (**D**) Non-specific interstitial pneumonia accompanied by mild alveolar epithelial hyperplasia.

The anatomopathological study revealed that *Dictyocaulus* induces bronchopneumonia in red deer, resulting from the presence of adult worms and larvae within the upper and middle respiratory tract. Figure 3A illustrates bronchiolar inflammation, the destruction of the bronchiolar epithelium (bronchiolitis), and an intraluminal exudate composed of eosinophils, lymphocytes, and plasma cells. Additionally, intraluminal parasitic structures (Figure 3B), identified as nematodes, were surrounded by extensive intraluminal hemorrhage and a mild peri-bronchial lymphoid reaction. Figure 3C highlights a distinct parasitic membrane composed of three structural components: a fibrous capsule (C), an acellular laminated membrane (L), and a germinative membrane (G). Figure 3D depicts a non-specific interstitial pneumonia characterized by diffuse mononuclear cell infiltration, which expands and thickens the interalveolar septa, with minimal collagenization and mild alveolar epithelial hyperplasia. No evidence of fibroblastic proliferation or panelization phenomena was observed.

### 3.3. Barcoding Findings Through Sequencing COI Gene

**Figure 4:**
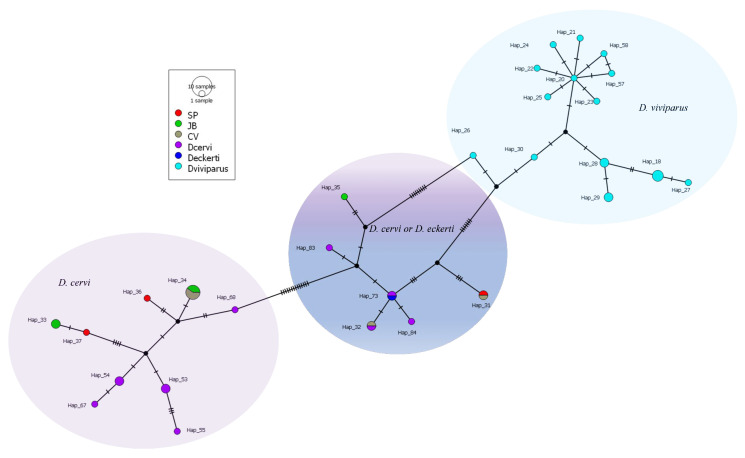
Graphical phylogenetic relationship among the three *Dictyocaulus* spp. genetic groups under a similarity threshold greater than 90% with respect to Genebank sequences (NCBI, USA). Central group consists of conflicting species assignations.

Barcoding was confidently conducted in three of the seven hunting enclosures, as adult worms were reliably identified in these locations. Consequently, San Fermín and Cuadrillas Bajas were excluded until future hunting seasons because no adult worms were observed in the former, and only the anatomopathological study was performed in the latter (see Table 2). In this study, gold-standard sequencing successfully yielded thirteen COI profiles, which collapsed into seven distinct haplotypes (Hap_31 to Hap_37, Figure 4). These haplotypes were compared with 102 additional sequences, which represent molecular data from twenty-four genera or species of nematode worms (Table S1). Globally, the 115 sequences collapsed in 84 unique haplotypes (Figure S1), with sequence similarities ranging between 97.0% and 99.6% compared to GenBank (NCBI, USA) entries. Exceptionally, one sequence from Cerro Verde (CV) showed 100% similarity but had a more uncertain species assignment, potentially belonging to *D. eckerti* (see Hap_32 vs. Hap_73, Figure 4).

Distinct haplotype clusters emerged, with most haplotypes aligning with taxonomic species reported in GenBank (Figure S1). Notably, the haplotype network clearly separated three species within the genus *Dictyocaulus*: *D. viviparus*, *D. eckerti*, and *D. cervi*. To enhance visualization, these species were analyzed separately (Figure 4). A dedicated network was constructed to exclusively examine haplotypes within the *Dictyocaulus* genus. This detailed analysis identified three distinct and well-differentiated matrilineal groups. Two groups contained haplotypes exclusively assigned to *D. viviparus* and D. cervi. The cluster linked to *D. cervi* consisted of Hap_33, Hap_34, Hap_36, and Hap_37. However, haplotypes belonging to *D. viviparus* were not found in this study. A third genetic group of admixed haplotypes encompassed closely related haplotypes previously assigned to either *D. cervi* or *D. eckerti* (Hap_31, Hap_32, and Hap_35) (Table S1). Since these haplotypes were designated to different species despite sharing a probable common ancestor distinct from *D. viviparus* or *D. cervi*, potential hybridization or taxonomic misassignment warrants further investigation.

Additionally, Hap_32 was the only haplotype with 100% identity to one of the GenBank sequences, which was originally assigned to *D. cervi* (accession No. PP922991) within the admixed group (Figure 4).

According to phylogenetic analysis (color assignment in the network, Figure 4), it should be noted that Hap_31 was found in SP (Sierra Palomares, n = 1) and CV (n = 1), Hap_32 in CV (n = 1), Hap_34 in both CV (n = 3) and JB (La Jabalina, n = 2), Hap_36/Hap_37 in SP (n = 1 each), and Hap_33/hap _35 in JB (n = 2 and n = 1, respectively) (Figure 4).

In addition to the phylogenetic evidence supporting cluster subdivisions, the AMOVA analysis further validated the genetic subdivision among the three *Dictyocaulus* groups, with a substantial proportion of genetic variance (89.8%) attributed to differences among groups—two of which showed clear species differentiation—while 10.2% of the variance was observed within groups, supported by a significant *ɸ_ST_* index (*p* < 0.001). These results support the matrilineal isolation of the *D. cervi* and *D. viviparus* haplotype groups, which align perfectly with the species assignment. In contrast, the admixed *D. cervi* and *D. eckerti* group shared a matrilineal ancestor within a third, genetically distinct group. As a result, the central gene cluster (Figure 4) does not conform to species assignment (see Table S1: haplotypes and species), suggestive of introgression. Moreover, haplotypes from the admixed group, as well as those uniquely associated with *D. cervi*, were identified across all sampling sites. Additionally, this evidence indicates the coexistence of *D. cervi* and *D. eckerti* within the same host. Specifically, Hap_31, Hap_32 (mixed group), and Hap_34 (*D. cervi* group) were simultaneously identified in the single lung sample obtained from CV. A similar pattern was observed at SP, where Hap_31, Hap_36, and Hap_37 were all found within a single lung sample.

## 4. Discussion

### 4.1. Prevalence of Infection by Dictyocaulus in Deer in Extremadura

Focusing on adult counts from macroscopic analysis, our results align with previous studies [14,36], which reported an average intensity of 11.7 and 6.3 adult worms per positive lung, respectively. However, substantial discrepancies were observed in other studies [5,37,38,39], which reported widely varying prevalence rates—from fewer than one adult per 27 positive lungs to all positive deer presenting adult worms. These variations likely stem from multifactorial influences, including environmental and physiological conditions, study design, and particularly the age and immune status of sampled animals. Differences in sample size may also play a role. Several authors [14,24,36,40,41,42,43] emphasized the importance of maximizing sample numbers, particularly in sampled locations.

From an epidemiological perspective, a prevalence of 22.2% was observed, closely matching previous findings in roe deer in northern Spain and deer in the Italian Alps [14,41]. In contrast, higher prevalence rates of 50% and 54% were reported in Norway [41,44], while studies in Poland [5,39] and Extremadura [24] recorded even greater prevalence rates—ranging from 44% to 68% and reaching 62.5% in red deer. These findings suggest that prevalence varies significantly over time and across geographic regions.

Furthermore, it would have been interesting to perform coprological analysis to determine how many larvae, if any, deer expel in their feces [24]. Despite this application of feces, given that genetically different worms can be found from the same lung (even with small samples), caution is advised when using conventional sequencing analysis based on genetic material obtained from feces. To ensure accuracy, adult worms or larvae should be individually analyzed when genetic studies are among the objectives, particularly in cases where hybridization is reasonably suspected. Moreover, our findings highlight the significance of individualized specimens, which contribute 44.44% (4/9) and 33.33% (2/6) of haplotypes to *D. cervi* and the admixed *D. eckerti*/*D. cervi* group, respectively, while making no contribution (0 from 14) to the *D. viviparus* group. Additionally, these results resemble those currently recorded in NCBI databases.

### 4.2. Findings After Anatomopathological Study

In agreement with some authors [5,28,45], morphological traits observed in worms may only assess their belonging to the genus *Dictyocaulus*. However, these traits can differentiate *Dictyocaulus* from other genera of pulmonary nematodes. Specifically, the adult specimens collected in our study exhibited a mouth surrounded by a well-pronounced cuticular ring, a long esophagus, and a bursa copulatrix with rays that allow its expansion during copulation. In males, the dark-colored spicules, shaped like a boot or sock, are guided by the gubernaculum, while in females, the uterus is filled with easily discernible larvated eggs. However, species-level identification using light microscopy is highly challenging as it relies on subjective measurements with minimal distinction among species. These limitations underscore the necessity for more standardized diagnostic methods to accurately identify *Dictyocaulus* species, such as molecular DNA methods. Furthermore, our findings introduce new and unexpected perspectives beyond the scope of traditional anatomopathological methods.

In the analysis of lung lesions caused by *Dictyocaulus* worms, both similarities and significant differences were identified compared to previous studies. Notably, our observations differ from those reported by other authors [39], as no substantial presence of macrophages was observed in the exudates analyzed. In addition, previous studies described extensive hyperplasia in follicular lymphoid follicles, bronchial vessels, and the bronchiolar epithelium, leading to a pronounced and widespread inflammatory and proliferative response in the affected lungs [39]. In contrast, only mild hyperplasia was detected in the alveolar epithelium, which may indicate a less intense inflammatory response across different stages of the alveolar epithelium. Another study [46] largely corroborated these findings, reinforcing the consistency of many pathological patterns associated with different dictyocaulosis scenarios. Also, congruence was observed with another study [47], except for some specific details. The presence of slight fibroblastic proliferation, necrotic changes, and the proliferation of type II pneumocytes has been observed, but these features were not prominently observed in the present study. These discrepancies may be attributed to environmental and/or genetic variations between different red deer populations. Additionally, differences in histological and sampling techniques cannot be ruled out. Overall, the data suggest a multifactorial and variable progression of infection, underscoring possible differential responses among *Dictyocaulus* species and/or species mixtures.

### 4.3. Molecular Assessment of Dictyocaulus spp.

Mitochondrial COI gene outcomes were consistent with the work of some authors [14,30] because relatively high molecular variability allowed for effective barcoding to discern all three *Dictyocaulus* species: *D. viviparus*, *D. eckerti*, and *D. cervi*. Of the eighteen nominal species within the genus, only four are reported to infect European deer species: *D. eckerti*, *D. capreolus*, *D. cervi*, and *D. skrjabini* [48]. Except *D. viviparus* from cattle, the remaining species in the genus have been found in sheep and goats (*D. filaria*), donkeys and horses (*D. arnfeldi*), camels (*D. cameli*), and African artiodactyls (*D. africanus*) and also, *D. skrjabini* were not present in our data set due to either their lower similarity threshold, different sequence coverage, or the absence of available data.

As a result, the downloaded molecular data only contained information from *D. viviparus*, reinforcing a close genetic relationship among these three species (*D. viviparus*, *D. eckerti*, and *D. cervi*) within the genus *Dictyocaulus*. Additionally, subspecies—or potentially new species—of *D. viviparus* have been suggested in both European [49] and North American bison [50], which were also in our data set (Hap_58 and Hap_57, Figure 4 and Table S1). However, it was not even possible to corroborate different lineage levels for these subspecies with the available data. All these findings support the high genetic variability and long-life evolutionary history of this genus. The global phylogenetic analysis (115 sequences) identified three major groups in the genus *Dictyocaulus*, characterized by substantial genetic variability between groups (89.9% variance) and less within groups (10.12% variance), which agrees with previous studies [5,14,39,50]. However, of the three genetic groups (*D. eckerti*, *D. cervi*, and *D. viviparus*), only the first two were found by us in red deer. Interestingly, this extensive genetic variability has been linked to the nematode’s possible adaptation to environmental changes, including the development of resistance to anthelmintics. Such resistance has been reported in a few studies [20,21,51]. The first study carried out in the same region of Extremadura identified infection with *D. viviparus* [24], though based on challenging morphological characters. However, the more refined barcoding-based approach in this study identified *D. cervi* and, probably, *D. eckerti* at this location. An MJN analysis revealed strict genetic relationships within lineages of *D. cervi* and *D. viviparus* sequences but allocating *D. eckerti* with several *D. cervi* haplotypes (specifically Hap_32), suggesting a mixed origin for this group (*D. eckerti*/*D. cervi* groups, Figure 4). These findings have also been reported previously [49]. The *ɸ_ST_* statistic supported a significantly high divergence among the three groups in this study, detecting strongly distinct mitochondrial COI alleles within the *Dictyocaulus* genus.

This study represents the first report of *D. eckerti* and *D. cervi* in Extremadura and Spain. In addition, our findings suggest a better and precise identification method of *Dictyocaulus* species, ruling out *D. viviparus* as the cause of *Dictyocaulus* infection in the examined red deer populations. According to some authors [5,14,39,45], there is firm evidence, even morphological, that *D. cervi* sampled from red deer may be well distinguished from *D. eckerti*, highlighting the importance of new COI sequences for diagnosis. Additionally, this study indicates the need for further research to clarify the presence of two species within one of the three genetic groups, especially when hybridization might be a plausible event, since in this study, both species were found in the same red deer lung. In this line of evidence, hybridization in nematode species has been demonstrated [52]. Also, hybridization, in which species share genetic material, may be influenced by environmental factors, host interactions, and life cycle similarities. Therefore, a more precise picture of the role of these multiple factors involved in complex host–parasite relationships has been called for, aiming at a broader and deeper understanding through an exploration of new regions and new methods [45] such as those implemented in our study.

## 5. Conclusions

The low prevalence of *Dictyocaulus* spp. suggests that the immune system of red deer plays a role in balancing these infections, as confirmed by the concordance between macroscopic and microscopic diagnoses. Molecular analysis revealed high genetic divergence among *Dictyocaulus* species for the first time in Spain. Notably, worms of different species were found within the same infected lung, underscoring the importance of integrating both morphological and molecular analyses for a more comprehensive understanding. Additionally, sequences attributed to different species within the same genetic group were identified, highlighting the need for further studies to distinguish between natural species mixtures and potential hybridization in these worms. So, our findings further support the idea that the numbers of species mixing in wild ruminants are greater than previously reported, calling for further investigation.

## Figures and Tables

**Table 1:** Location and date of material collection.

Name	Location	Date
Cuadrillas Bajas	Cedillo	21/09/23
San Fermín	Torrejón el Rubio	03/12/23
Sierra Palomares	Alía	27/01/24
Jabalina	Salorino	02/02/24
Valdelayegua	Aliseda	09/02/24
Cerro Verde	Carbajo	11/02/24
El Águila	Serradilla	17/02/24

**Table 2:** Number of red deer, location, macroscopic and microscopic results, and adult worms found. + = presence; - = absence; nf = not found.

Lungs	Location	Macroscopic	Microscopic	Adult Number
Deer 1	Cuadrillas Bajas	+	+	4
Deer 2	Cuadrillas Bajas	-	+	nf
Deer 3	Cuadrillas Bajas	-	-	nf
Deer 4	Cuadrillas Bajas	-	-	nf
Deer 5	Cuadrillas Bajas	-	-	nf
Deer 6	San Fermín	-	-	nf
Deer 7	San Fermín	-	-	nf
Deer 8	San Fermín	-	-	nf
Deer 9	San Fermín	-	+	nf
Deer 10	San Fermín	-	-	nf
Deer 11	San Fermín	-	-	nf
Deer 12	Sierra Paloma	-	-	nf
Deer 13	Sierra Paloma	-	-	nf
Deer 14	Sierra Paloma	-	-	nf
Deer 15	Sierra Paloma	-	+	nf
Deer 16	Sierra Paloma	+	+	8
Deer 17	Jabalina	+	+	17
Deer 18	Jabalina	+	+	22
Deer 19	Jabalina	-	-	nf
Deer 20	Jabalina	-	-	nf
Deer 21	Jabalina	-	-	nf
Deer 22	Valdelayegua	-	-	nf
Deer 23	Valdelayegua	-	-	nf
Deer 24	Valdelayegua	-	-	nf
Deer 25	Valdelayegua	-	-	nf
Deer 26	Valdelayegua	-	-	nf
Deer 27	Cerro Verde	-	-	nf
Deer 28	Cerro Verde	-	-	nf
Deer 29	Cerro Verde	-	-	nf
Deer 30	Cerro Verde	-	-	nf
Deer 31	Cerro Verde	+	+	13
Deer 32	El Águila	-	-	nf
Deer 33	El Águila	-	-	nf
Deer 34	El Águila	-	-	nf
Deer 35	El Águila	-	-	nf
Deer 36	El Águila	-	-	nf
TOTAL		5	8	64

## Data Availability

Data are available on request to the authors, though the sequences have been deposited in the GeneBank database (NCBI, USA) under the accession numbers PV700498–PV700500.

## Supplementary Materials

The following supporting information can be downloaded at https://www.mdpi.com/article/10.3390/vetsci12060595/s1, Figure S1: Graphical representation of the distribution of the 81 different haplotypes used for MJN analysis. Colors were selected according to assigned species in GenBank report. Sampling sites are from this study and others on non-*Dictyocaulus* genera; Table S1: Species of worms from Genbank (NCBI, USA) with a higher than 90% threshold similarity.
