# Supplementary material for: Contributions to Knowledge of the Dictyocaulus Infection of the Red Deer
Source: Vet Sci. 2025 Jun 17;12(6):595. doi: 10.3390/vetsci12060595 (PMC12197584; doi:10.3390/vetsci12060595)
Supplement: Supplementary file 1 [file vetsci-12-00595-s001.zip › vetsci-3602307-supplementary.pdf]

Supplementary Materials:

Table S1. Species of worms from genbank (NCBI, USA) with a higher 90% threshold similarity.

Acc. Number	Species ID	Haplotype figure S1
>AB682712	*Spiculopteragia choudemeri*	Hap_1
>AB682713	*Spiculopteragia houdemeri*	Hap_2
>AB682724	*Spiculopteragia houdemeri*	Hap_3
>AB682726	*Spiculopteragia houdemeri*	Hap_4
>AB682727	*Spiculopteragia houdemeri*	Hap_5
>AB682728	*Spiculopteragia houdemeri*	Hap_6
>AB793548	*Necator sp.*	Hap_7
>AB793549	*Necator sp.*	Hap_8
>AB793551	*Necator sp.*	Hap_9
>AB793552	*Necator sp.*	Hap_10
>AB793553	*Necator sp.*	Hap_11
>AB793554	*Necator sp.*	Hap_12
>AB793555	*Necator sp.*	Hap_10
>AB793556	*Necator sp.*	Hap_13
>AB793557	*Necator sp.*	Hap_14
>AB793558	*Necator sp.*	Hap_15
>AB793559	*Necator sp.*	Hap_13
>AB793560	*Necator sp.*	Hap_13
>AB793561	*Necator sp.*	Hap_16

Acc. Number	Species ID	Haplotype figure S1
>AB793562	*Necator sp.*	Hap_12
>AB793563	*Necator sp.*	Hap_17
>AF263474	*Dictyocaulus viviparus*	Hap_18
>AF263486	*Strongylus equinus*	Hap_19
>AJ430568	*Dictyocaulus viviparus*	Hap_20
>AJ430569	*Dictyocaulus viviparus*	Hap_21
>AJ430570	*Dictyocaulus viviparus*	Hap_22
>AJ430571	*Dictyocaulus viviparus*	Hap_23
>AJ430572	*Dictyocaulus viviparus*	Hap_24
>AJ430573	*Dictyocaulus viviparus*	Hap_25
>AJ430574	*Dictyocaulus viviparus*	Hap_26
>AJ430575	*Dictyocaulus viviparus*	Hap_18
>AJ430576	*Dictyocaulus viviparus*	Hap_27
>AJ430577	*Dictyocaulus viviparus*	Hap_28
>AJ430578	*Dictyocaulus viviparus*	Hap_29
>AJ430579	*Dictyocaulus viviparus*	Hap_30
>AP017683	*Dictyocaulus viviparus*	Hap_28
>EU628683	*Baylisascariasis transfuga*	Hap_38
>EU740387	*Baylisascariasis transfuga*	Hap_38

Acc. Number	Species ID	Haplotype figure S1
>HM594948	*Baylisascariasis transfuga*	Hap_38
>KC543477	*Baylisascariasis transfuga*	Hap_38
>KJ851946	*Cloacina robertsi*	Hap_39
>KJ851947	*Cloacina robertsi*	Hap_40
>KJ851948	*Cloacina robertsi*	Hap_41
>KJ851949	*Cloacina robertsi*	Hap_42
>KP876338	*Murielus harpespiculus*	Hap_43
>KP876339	*Murielus harpespiculus*	Hap_44
>KP876340	*Murielus harpespiculus*	Hap_45
>KP876341	*Murielus harpespiculus*	Hap_46
>KP876342	*Murielus harpespiculus*	Hap_46
>KP876351	*Murielus harpespiculus*	Hap_47
>KP876354	*Murielus harpespiculus*	Hap_48
>KP876362	*Murielus harpespiculus*	Hap_49
>KP876374	*Murielus harpespiculus*	Hap_50
>KP876375	*Murielus harpespiculus*	Hap_51
>KP876376	*Murielus harpespiculus*	Hap_52
>KR231672	*Dictyocaulus cervi*	Hap_53
>KR231674	*Dictyocaulus cervi*	Hap_53

Acc. Number	Species ID	Haplotype figure S1
>KR231675	*Dictyocaulus cervi*	Hap_54
>KR231676	*Dictyocaulus cervi*	Hap_55
>KT262937	*Strongylus equinus*	Hap_56
>KT581635	*Dictyocaulus viviparus bisontis*	Hap_57
>KT581636	*Dictyocaulus viviparus bisontis*	Hap_58
>KT984867	*Strongylus edentatus*	Hap_59
>KY365437	*Perostrongylus falciformis*	Hap_60
>KY973960	*Baylisascariasis transfuga*	Hap_61
>LC088303	*Necator sp.*	Hap_62
>LC088310	*Necator sp.*	Hap_63
>LC088312	*Necator sp.*	Hap_9
>LC088313	*Necator sp.*	Hap_12
>LC088314	*Necator sp.*	Hap_11
>LC088321	*Necator sp.*	Hap_64
>MF419818	*Baylisascariasis transfuga*	Hap_65
>MH795152	*Baylisascariasis schroederi*	Hap_38
>MK558921	*Baylisascariasis transfuga*	Hap_66
>MT914191	*Dictyocaulus cervi*	Hap_67
>MT914192	*Dictyocaulus cervi*	Hap_54

Acc. Number	Species ID	Haplotype figure S1
>MT914266	*Dictyocaulus cervi*	Hap_68
>MW026408	*Baylisascariasis transfuga*	Hap_64
>MW309877	*Macropostrongyloides lasiorhini*	Hap_69
>MW517832	*Arthrostoma sp.*	Hap_70
>MW843794	*Elaphostrongylus rangiferi*	Hap_71
>MZ665481	*Ancylostoma sp.*	Hap_72
>NC15924	*Baylisascariasis transfuga*	Hap_38
>NC19809	*Dictyocaulus eckerti*	Hap_73
>NC19810	*Dictyocaulus viviparus*	Hap_29
>NC23262	*Protostrongylus rufescens*	Hap_74
>NC67627	*Zoniolaimus dendrolagi*	Hap_75
>NC67629	*Parazoniolaimus collaris*	Hap_76
>NC68834	*Varestrongylus eleguneniensis*	Hap_77
>OK111103	*Oesophagostomoides stirtoni*	Hap_78
>OK111105	*Paramacropostrongylus iugalis*	Hap_79
>OK111106	*Paramacropostrongylus iugalis*	Hap_79
>OK111108	*Torquenema toraliforme*	Hap_80
>ON982732	*Baylisascariasis laevis*	Hap_81
>OP602952	*Lagochilascaris minor*	Hap_82

Acc. Number	Species ID	Haplotype figure S1
>OP602953	*Lagochilascaris minor*	Hap_82
>OP617688	*Dictyocaulus cervi*	Hap_83
>OR131278	*Baylisascariasis transfuga*	Hap_38
>OZ076388	*Dictyocaulus viviparus*	Hap_18
>PP922991	*Dictyocaulus cervi*	Hap_32
>PP922995	*Dictyocaulus cervi*	Hap_73
>PP923005	*Dictyocaulus cervi*	Hap_84

Figure S1. Graphical representation of the distribution of the different 81 haplotypes used for MJN analysis. Color were selected according to assigned species in Genebank report, sampling sites when form those study and others from non Dictyocaulus genus.

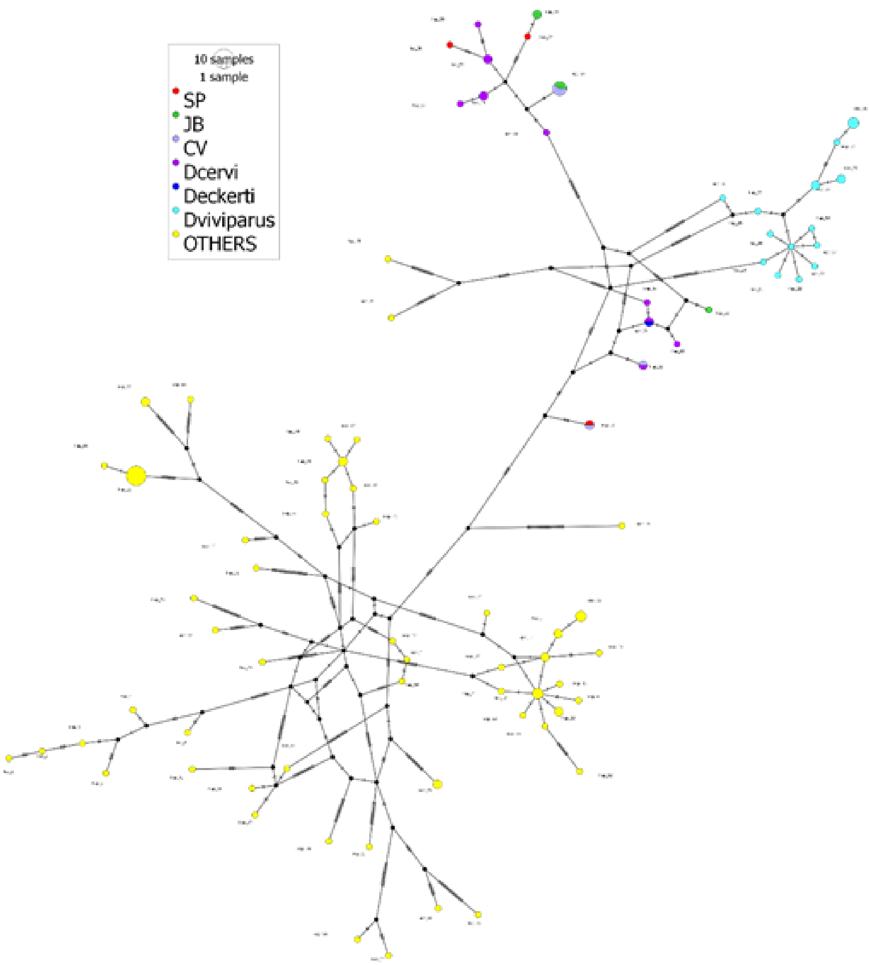
10 samples
1 sample
SP
JB
CV
Dcervi
Deckerti
Dviviparus
OTHERS
